# Update on the practice of premarital screening for sickle cell traits in Africa: a systematic review and meta-analysis

**DOI:** 10.1186/s12889-024-19001-y

**Published:** 2024-05-31

**Authors:** Priscilla Peter Dilli, Emmanuel Obeagu, Andrew Tamale, Anselm Ajugwo, Theophilus Pius, Danladi Makeri

**Affiliations:** 1 Department of Public Health, Kampala International University-Western Campus, Ishaka, Uganda; 2 Department of Medical Laboratory Science, Kampala International University-Western Campus, Ishaka, Uganda; 3 Department of Microbiology and Immunology, Kampala International University-Western Campus, Ishaka, Ishaka Uganda

**Keywords:** Sickle cell disease, Sickle cell trait screening, Premarital genotype screening, Premarital Genetic Counseling, Preconception Genetic Screening, Hemoglobinopathy

## Abstract

**Background:**

Screening for sickle cell traits before marriage or producing children is one of the outstanding preventive measures for sickle cell disease (SCD).The disease is a collection of inherited blood disorders that impact millions globally, with a predominant 75% occurrence in the sub-Saharan region. With increasing burden of SCD on the continent amidst a cost effective prevention method, no study has systematically reviewed or presented meta-analytic uptake or practice of premarital sickle cell trait screening.

**Methods:**

This review systematically explored the uptake or practice of premarital genotype screening in Africa. We searched PubMed and Scopus databases for African studies on premarital screening for sickle cell traits.

**Results:**

Our results indicate that the pooled uptake of premarital sickle cell trait screening in Africa is 47.82% (95% CI: [46.53–49.11]; I^2^: 98.95% [98.74–99.13]). Our review observed, a significant relationship between the awareness of sickle cell disease and the uptake of genotype screening; F(1, 13) = 12.04, *p* = 0.004). The model explained approximately 48.08% of the variation in genotype screening (R² = 0.4808) and predicted a 0.729 increase in the likelihood of genotype screening uptake for every unit rise in sickle cell disease awareness (β = 0.729, *p* = 0.004). Additionally, Pearson correlation (*r* = 0.6934) indicated a moderately strong positive correlation between the two variables.

**Conclusion:**

With over 75% of the global burden of sickle cell disease domiciled in Africa, the continent cannot overlook the cost of hemoglobinopathies. The uptake of sickle cell traits screening is suboptimal across the continent. To achieve the mandate of sustainable development goal number (3); to end preventable deaths of newborns and children under 5 years of age by 2030, there is need to intensify campaigns on premarital genetic screening through education and other health promotion tools.

**Supplementary Information:**

The online version contains supplementary material available at 10.1186/s12889-024-19001-y.

## Background

Millions of people worldwide are affected by sickle cell disease (SCD) [1] and more than 75% of global cases occur in sub-Saharan Africa [2]. In this region, SCD poses a significant public health burden, leading to increased mortality rates and reduced quality of life for affected individuals [3].

Theoretically, sickle cell traits, also known as sickle cell carrier genes, are transmitted from parents to offspring [4]. When two partners carrying the trait decide to produce, there is a 25% probability of producing a sickle cell-diseased child in each pregnancy; the chances double when one partner is sickle-celled, and the other is a carrier [5]. Preventing the birth of children with SCD is a critical public health goal, and one way to achieve this is through testing for the heamoglobinopathy trait before producing children (preconception screening) or before marriage(premarital screening) [6]. Another mechanism of prevention is prenatal screening, a procedure conducted during pregnacy on the mother to determine if she carries the sickle cell trait or if she has sickle cell disease [7]; or on the fetus through amniocentesis [8].

Premarital sickle cell trait screening uptake vary widely among countries and regions. In some countries, such as Nigeria, premarital screening is taken seriously, and intending couples in certain faiths must show proof of screening before getting married [9]; this is not the same in Uganda, where most partners do not know their genotypes. Also, early sexual debut resulting in a high prevalence of teenage pregnancies is a challenge to preconception sickle cell trait screening (SCTS) in Uganda [10].

With several studies across Africa investigating knowledge, attitude, and practices toward SCTS and SCD, no study has systematically reviewed or presented meta-analytic uptake or practice of premarital genotyping, a cost effective prevention tool for SCD in Africa; thus, the basis for this review.

## Main text

### Method

#### Search strategy

We searched PubMed and Scopus databases from inception through 26th of March 2023 for African studies which captured premarital SCTS. We formulated our search query by combining key concepts related to the study as presented in Table 1. Gray literature and other studies published in journals not indexed in the PubMed and Scopus were retrieved from Google scholar.

#### Study selection criteria

**Fig. 1:**
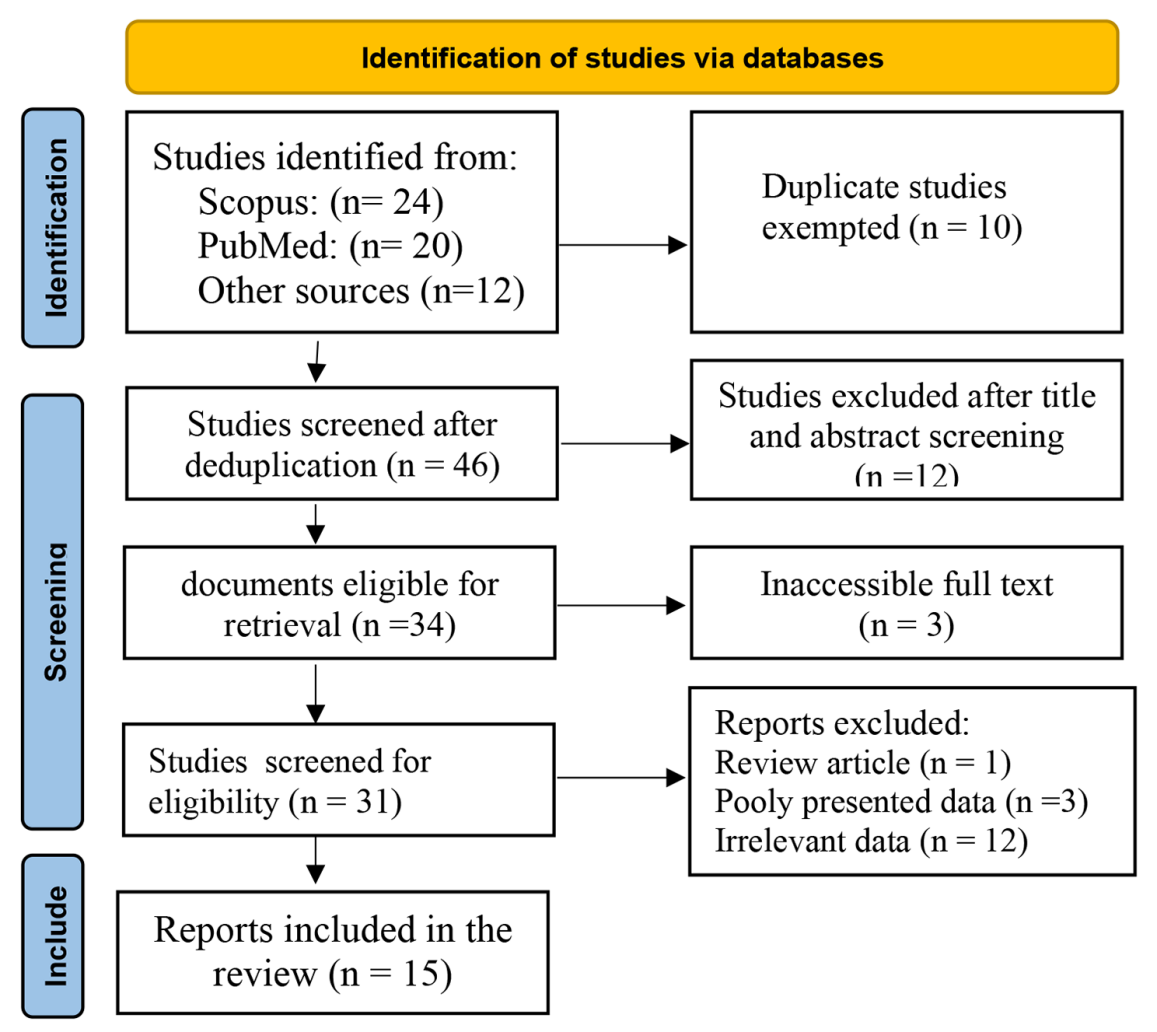
Study selection flowchart

We included studies conducted in Africa, among unmarried respondents. Studies that recruited mainly married respondents were excluded because the focus of this review is on premarital screening practices. We also excluded studies without accessible full texts to ensure the ability to assess the quality and details of the methodologies used, as incomplete data could compromise the validity of the review findings [11]. Meta-analyses, review articles, and phenomenological studies were excluded to avoid redundancy, focus on primary research data, and because phenomenological studies provide qualitative data that are not suitable for the quantitative synthesis methods used in this systematic review and meta-analysis. The study adhered to the Preferred Reporting Items for Systematic Reviews and Meta-analysis [12] (Fig. 1).

**Table 1:** Search Query

Database	Search query
Scopus & PubMed	(“premarital”) AND (“sickle cell screening” OR “Haemoglobin Genotype” OR “Genetic screening” OR “Genetic counseling”) AND (“Malawi” OR “Gambia” OR “Central African Republic” OR “Burkina Faso” OR “Kenya” OR “Ghana” OR “Mali” OR “Zambia” OR “Comoros” OR “Cabo Verde” OR “Mauritius” OR “Senegal” OR “Nigeria” OR “Liberia” OR “Equatorial Guinea” OR “Namibia” OR “Tanzania” OR “Rwanda” OR “Libya” OR “Ivory Coast” OR “Angola” OR “Seychelles” OR “Egypt” OR “South Sudan” OR “Ethiopia” OR “Botswana” OR “Sudan” OR “Djibouti” OR “Sierra Leone” OR “Morocco” OR “Madagascar” OR “Eswatini” OR “Uganda” OR “Tunisia” OR “Guinea-Bissau” OR “Chad” OR “Benin” OR “Burundi” OR “Lesotho” OR “Zimbabwe” OR “Guinea” OR “Cameroon” OR “Niger” OR “South Africa” OR “DR Congo” OR “Gabon” OR “Algeria” OR “Sao Tome and Principe” OR “Mauritania”)

### Data extraction

Data extraction, deduplication, and title and abstract screening were done independently by two authors (DM and PPD). DM and EO accessed the full text of studies which passed title and abstract screening and screened them for eligibility of inclusion criteria. We created a standardized Microsoft Excel (2019) spreadsheet into which we extracted and added relevant data from included studies into columns labeled as follows: author, number of people recruited into the study, uptake of SCTS, awareness of SCD, period of study, study design, publication year and country of study as presented in Table 2 below.

**Table 2:** Included Study Characteristics

Authors	Year	Country	Sample size	Study Pop.	Females	Males	Premarital Uptake of SCTS	Premarital SCD Awareness	Muslim	Christians	Study Design	Study Period
Agbozo et al. [13]. ,	2023	Ghana	451	Females	451	0	162	321	NR	NR	CR	2020
Oluwole et al. [14]. ,	2022	Nigeria	300	Both	122	188	129	139	64	236	CR	2019
Ameen et al. [15]. ,	2016	Nigeria	372	Both	186	186	105	346	39	66	CR	2014
Olakunle et al. [16]	2013	Nigeria	137	Both	67	70	81	131	12	121	CR	NR
Alao et al. [17]. ,	2009	Nigeria	300	Both	120	180	141	247	15	129	CR	2008
Abioye-Kuteyi et al. [18]. ,	2009	Nigeria	300	Both	171	129	260	93	NR	NR	CR	NR
Kisakye et al. [19]. ,	2022	Uganda	315	Both	122	193	77	295	23	292	CR	2021
Moronkola & Fadairo [20],	2006	Nigeria	783	Both	393	390	600	602	0	783	CR	2003
Ngwengi et al. [21]. ,	2020	Cameroun	410	Both	211	199	48	84	2	408	CR	2017
Kanma-Okafor et al. [22]. ,	2022	Nigeria	300	Both	190	110	196	189	147	153	CR	NR
Oluwadamilola et al. [23]. ,	2021	Nigeria	420	Both	239	189	230	246	180	240	CR	NR
Bademosi [24],	2016	Nigeria	377	Both	229	148	322	336	47	325	CR	NR
Tusuubira et al. [25]. ,	2018	Uganda	110	Both	63	39	20	93	NR	NR	CR	2016
Arthur & Koffuor [26],	2022	Ghana	405	Both	148	257	145	251	54	342	CR	2021
Bazuaye et al. [27]. ,	2009	Nigeria	850	Both	518	332	272	153	17	807	CR	2007

**Key**: * CR = Cross-sectional; *NR = Not Reported; *SCD = Sickle Cell Disease; *SCTS = Sickle Cell Trait Screening

### Critical appraisal

The Joanna Briggs Institute (JBI) Critical Appraisal Checklist for studies reporting prevalence [11] was used to assess the quality and risk of bias of included studies. Two authors (MD and PPD) independently performed the appraisals; whenever there was a discrepancy, it was resolved by consensus using the nominal group technique [28]. The result of the critical appraisal is presented in the Table 3 below.

**Table 3:** Risk of Bias Assessment

Study	Q1	Q2	Q3	Q4	Q5	Q6	Q7	Q8	Q9	Score
Abioye-Kuteyi et al. [18]. ,	Y	Y	Y	Y	Y	Y	Y	Y	Y	100
Agbozo et al. [13]. ,	Y	Y	Y	Y	Y	Y	Y	U	Y	89
Alao et al. [17]. ,	Y	U	Y	Y	Y	Y	Y	Y	Y	89
Ameen et al. [15]. ,	Y	Y	Y	Y	Y	Y	Y	Y	Y	100
Arthur & Koffuor [26],	Y	Y	Y	Y	Y	Y	Y	Y	Y	100
Bademosi [24],	Y	Y	Y	Y	Y	Y	Y	Y	Y	100
Bazuaye et al. [27]. ,	Y	U	Y	Y	Y	Y	Y	Y	Y	89
Kanma-Okafor et al. [22]. ,	Y	U	Y	Y	Y	Y	Y	Y	Y	89
Kisakye et al. [19]. ,	Y	Y	Y	Y	Y	Y	Y	Y	Y	100
Moronkola & Fadairo [20],	Y	Y	Y	Y	Y	Y	Y	U	Y	89
Ngwengi et al. [21]. ,	Y	Y	Y	Y	Y	Y	Y	Y	Y	100
Olakunle et al. [16]. ,	Y	Y	Y	Y	Y	Y	Y	Y	Y	100
Oluwadamilola et al. [23]. ,	Y	Y	Y	Y	Y	Y	Y	U	Y	89
Oluwole et al. [14]. ,	Y	Y	Y	Y	Y	Y	Y	Y	Y	100
Tusuubira et al. [25]. ,	Y	Y	Y	Y	Y	Y	Y	Y	Y	100
Total										95.6%

KEY: *Q = Question; *Y = Yes; *U = Unclear.

Q1: Was the sample frame appropriate to address the target population?

Q2: Were study participants sampled in an appropriate way?

Q3: Was the sample size adequate for this analysis?

Q4: Were the study subjects and the setting described in detail?

Q5: Was the data analysis conducted with sufficient coverage of the identified sample?

Q6: Were valid methods used for the evaluation of the uptake of SCTS?

Q7: Was the Uptake of SCTS evaluated in a standard, reliable way for all participants?

Q8: Was there appropriate statistical analysis?

Q9: Was the response rate adequate, and if not, was the low response rate managed appropriately?

### Statistical analysis

We used the random effect model to calculate the uptake of SCTS at 95% confidence intervals and I^2^ statistics to assess study heterogeneity. I^2^ at 95%CI was interpreted as low, moderate, or high values (≤ 25%), (25–75%), and (≥ 75%), respectively [29]. All meta-analyses were performed using R statistical package.

## Results

### Study selection and characteristics

A systematic search of PubMed and Scopus databases retrieved 44 studies; twelve (12) other studies not indexed in these databases were retrieved from Google search making a total of 56 studies. Ten (10) duplicates were removed, followed by twelve (12) ineligible titles and abstracts.Another 3 studies with inaccessible full text were excluded. Thirty-one (31) studies were screened for eligibility of full text and all components of the inclusion criteria. At this stage, (1) review article, twelve (12) studies lacking relevant data, and (3) studies with inadequate data were excluded, as shown in Fig. (1) below. A critical appraisal of the eligible studies showed that a negligible portion of the respondents were married. There were instances when studies only mentioned statistical packages used for analysis without mentioning the particular statistics used. In studies with some married respondents, we extracted the uptake of SCTS reported for unmarried respondents. These discrepancies were, however, not considered grounds for exclusion.

A total of fifteen (15) cross-sectional studies conducted between 2003 and 2021 were included in this review. The studies, spread across western and eastern Africa, including Nigeria, Cameroun, Uganda, and Ghana recruited a total of 5380 respondents spread across two genders and religions. Three thousand, two hundred and thirty (3230) respondents constituting 55.4%, were females while 67% of the respondents were Christians.

### Uptake/practice of sickle cell trait screening across Africa

**Fig. 2:**
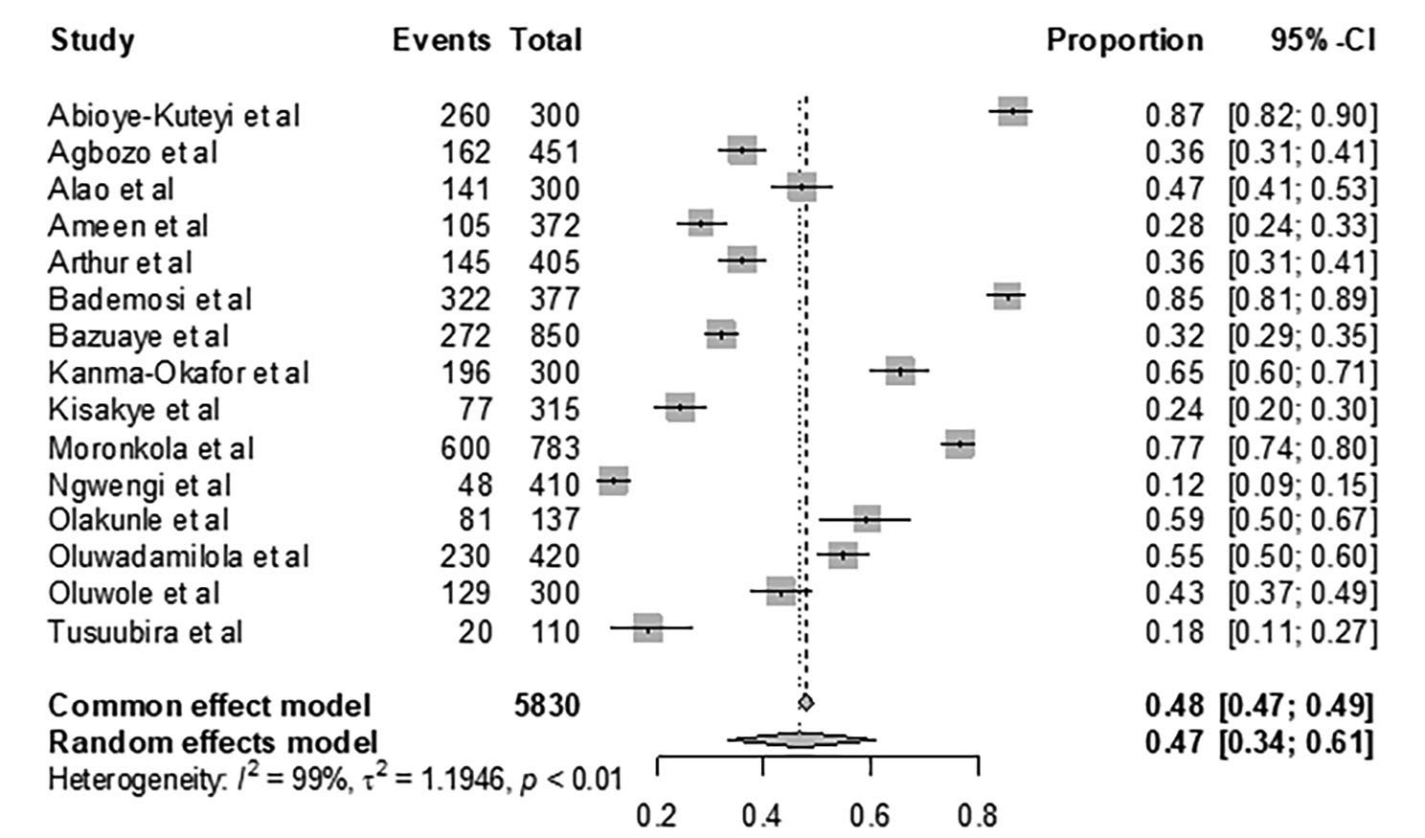
Forest Plot of Pooled Proportion of Premarital SCTS Uptake in Africa

Sub-Saharan Africa has 75% of the global burden of SCD. Preventing the birth of children with SCD is a critical public health goal, and one way to achieve this is through screening for hemoglobin S-related illness traits before marriage or producing children. Our meta-analysis observed a continental pooled SCTS uptake of 47.822% (95% CI: [46.53–49.11]; I^2^: 98.95% [98.74–99.13]). Across the different studies, the uptake of SCTS ranged from as high as 86.67% (95% CI: [82.29–90.30]; I^2^: 99.02% [98.82–99.19]) to as low as 11.72% (95% CI: [8.76–15.22]). Figure 2 summarizes SCTS uptake across Africa.

### Awareness of sickle cell disease in Africa

**Fig. 3:**
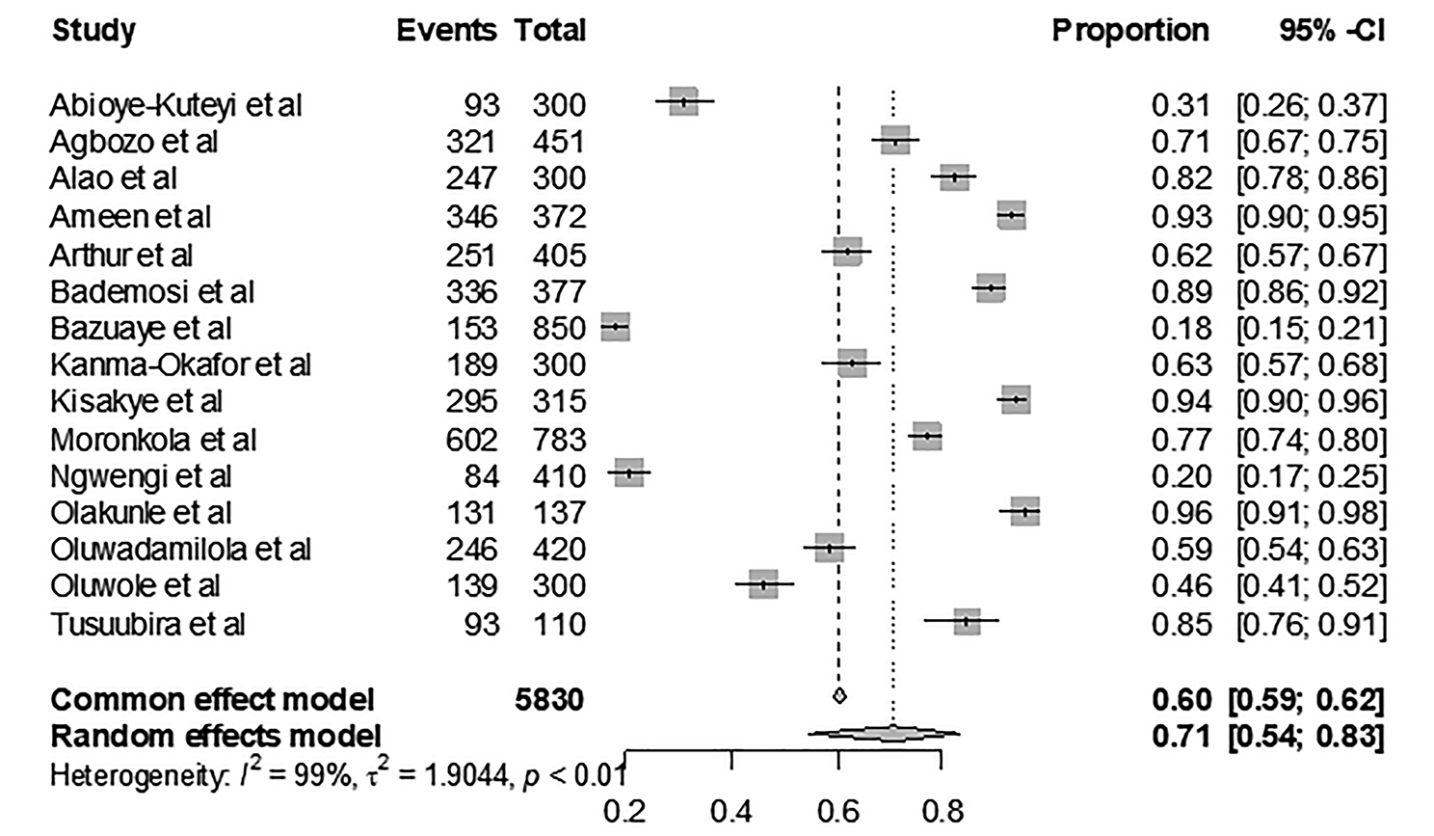
Forest Plot of Sickle Cell Disease Awareness in Africa

Uptake or practice of health behaviors has been attributed to individuals’ perception of susceptibility and knowledge of the condition in question. In regards to sickle cell trait screening, this meta-analysis sought to understand the level of awareness of SCD in Africa. The forest plot in Fig. 3 below presents the proportions from the different studies across Africa.

### Country-wise uptake of genotype screening in Africa

Table 4 below presents a subgroup meta-analytic pooled prevalence of SCTS uptake in different African countries. In Cameroon, for instance, based on 410 respondents, the practice of SCTS was 11.71% (8.76–15.22). The analysis showed high heterogeneity, with an I^2^ value of 99.48% (99.28–99.62). Our meta-analysis observed varying levels of SCTS uptake across different African countries.

**Table 4:** National Uptake of Sickle Cell Disease in Africa

Country	Sample size	Proportion (%)	95% CI	Weight	I^2^ (95%CI)	*P*-value
Camero on	410	11.71	8.76–15.22	24.9	99.48% (99.28–99.62)	< 0.0001
Ghana	856	35.86	32.65–39.18	25.04		
Nigeria	4139	56.44	54.91–57.96	25.15		
Uganda	425	22.82	18.92–27.11	24.91		

*CI = Confidence Interval, *I^2^ = Measure of heterogeneity, % = Percentage.

### Relationship between SCTS uptake and SCD awareness

We performed a linear regression analysis to assess the relationship between awareness of hemoglobin S-related illness and uptake of the associated trait screening. Our analysis revealed a significant relationship between the awareness of SCD and the uptake of SCTS F(1, 13) = 12.04, *p* = 0.004). The model explained approximately 48.08% of the variation in SCTS uptake (R² = 0.4808). An uptick of one unit in SCD awareness showed a corresponding rise of 0.729 in the odds of adopting SCTS (β = 0.729, *p* = 0.004). Our analysis suggests that persons with more knowledge of hemoglobin S-related illness are more likely to undergo genotype screening. Pearson correlation (r) for the relationship was 0.6934.

## Discussion

Our study aimed to assess the extent of premarital sickle cell trait/genotype screening in Africa among Africans. Our findings revealed a varied uptake of screening practices across different regions in Africa. Notably, the pooled proportion of SCTS uptake in Africa was estimated to be 47.82%(95% CI: [46.53–49.11; I^2^: 98.95% [98.74–99.13]), with individual study proportions ranging from 11.72 to 86.67%. This diversity can be attributed in one part to the national and regional differences on the burden of SCD. Adigwe et al. [30]. , reported that majority (74.5%) of studies on the burden of SCD in Africa are conducted in Nigeria; alligning with the country’s observed dorminance in SCTS uptake. Moreover, studies on SCD from other African countries are generally low thus impacting the promotion of premarital sickle cell trait screening. However, premarital SCTS can be impacted by a number of factors including awareness, age, availability and cost of screening services, individual perception of SCD, traditional beliefs, and family history of sickle cell disease [21, 31, 32].

Our meta-analysis also explored the level of awareness of SCD in Africa to corroborate the uptake of SCT. The pooled prevalence of SCD awareness was variable across the different studies (Fig. 3). In examining the relationship between SCTS uptake and SCD awareness, a regression analysis revealed a significant positive association. For every unit increase in SCD awareness there is a corresponding 0.729 increase in the likelihood of an individual screening for SCT (β = 0.729, *p* = 0.004). The model explained approximately 48.08% of the variation in SCTS uptake (R² = 0.4808). This observation alligns with the study by Agofure & Danzaria [31], where 58.6% of their respondents had poor knowledge of SCD and 34.6% said that SCTS is a waste of time.

A subgroup meta-analysis of the uptake of SCTS among African countries also indicated considerable variations. Cameroon was observed to have the lowest 11.71% (95% CI: 8.76–15.22; I^2^: 99.48% [99.28–99.62]) uptake of SCTS, while Nigeria had the highest. This analysis also showed a high level of heterogeneity with statistically significant (< 0.001) variation among the included studies. While we have previously observed a relationship between SCD awareness and SCTS, the reverse is the case in Cameroon, where the respondents had a considerable knowledge of SCD, yet the practice of genotype screening was poor [21]. The lack of translation of knowledge of SCD to uptake of genotype screening may be linked to socioeconomic factors such as poverty and poor access to screening services. The translation of knowledge (awareness) to the uptake of services will require commitment including, legally mandated premarital hemoglobinopathy testing [17] or educational campaigns with free or incentivized screening.

In the Nigerian context, the high uptake of genotype screening observed speaks to several issues including, ranking first on the burden of SCD worldwide [30] and the role of faith-based organizations. Faith-based organizations, particularly among Christians, mandate intending couples to go for genotype tests and may refuse to solemnize such unions when results are not favourable [9]. We performed a regression analysis to investigate the relationship between SCTS uptake and religion; the coefficients for the independent variables (religious affiliations) indicate a change in the log odds of SCTS uptake associated with each unit increase in the respective predictor variable. For Christians, the coefficient is 0.4797, with a standard error of 0.1565 and a significant t-value of 3.066 (*p* = 0.0134). This suggests that being a Christian is associated with a positive effect on the likelihood of SCTS uptake. Conversely, for Muslims, the coefficient is 0.6509, with a non-significant t-value of 0.984 (*p* = 0.3507), indicating that there is no significant relationship between being Muslim and the uptake of SCTS. Although we observed a positive relationship with being a Christain; the relationship may be biased because most of the respondents recruited into the various studies identified as Christians.However, it is worthy of note that in countries like Nigeria, churches have been reported to take SCTS seriously and often admonish their members to screen. While the churches’ approach has proven beneficial, it presents several ethical issues [9]. In Nigeria, we also observed a gross lack of literature from the northern region, which is densely populated with predominantly Muslims.

In Uganda, the pooled uptake of genotype screening is 22.82% (95% CI: [18.92–27.1]). The country currently ranks fifth in the global burden of sickle cell births and the highest in the East African Subcontinent [33]. The high number of children born with sickle cell disease in Uganda can partly be attributed to the high level of teenage pregnancies, estimated at 25% and the highest in East Africa [34]. These pregnancies directly impact the sickle cell burden because they are unplanned and occur when both teenagers are unaware of genetic diseases. In one study among 480 teenage girls in the Lira district in Eastern Uganda, 90.3% reported their first sexual encounter at ages 15–19 years [35], underscoring the role of early sexual debut in unwanted teenage pregnancies and sickle cell disease prevalence. While teenage pregnancies resulting from early sexual debut may be a key player in the Uganda sickle cell disease surge, a lack of awareness of the disease cannot be ruled out. In another study in Lubaga, Kampala, central Uganda, among 110 respondents, 44.2% had no knowledge of the cause of sickle cell disease. There is a need to intensify awareness campaigns about SCD and SCTS in this part of the continent. However, in Uganda, genotype screening before producing children should be emphasized against screening before marriage because 20–30% of Ugandan women produce children without marrying; this is the case in Kenya [36, 37].

While our study provides valuable insights into the uptake of premarital sickle cell trait screening across Africa, there are limitations to consider. First, significant heterogeneity was observed among the included studies, which may be attributed to variations in population characteristics and cultural factors across the continent. Although we used a random effects model to account for heterogeneity, it is important to acknowledge that the pooled estimates may still be influenced by these differences.Furthermore, the paucity of eligible studies for inclusion with more than half of the studies conducted in specific regions and countries (West Africa, and Nigeria) poses a potential limitation to the generalizability of our findings. Despite these limitations, our research contributes to the existing knowledge base and highlights the need for further investigations to address these challenges and improve screening uptake in Africa.

## Conclusions

We observed variations in sickle cell traits screening uptake across different countries and populations of Africa. Our findings confirm that uptake of genotype screening is linked to sickle cell disease awareness and highlight the need to intensify awareness campaigns. Future research should identify the barriers and facilitators influencing SCTS uptake and develop strategies to improve awareness and access to screening services. Addressing these factors may promote the uptake of genotype screening thus reducing the burden of SCD in Africa and achieving the sustainable development goal number three (3) which aims to end preventable deaths of newborns and children under 5 years of age by 2030.

### Electronic supplementary material

Below is the link to the electronic supplementary material.

Supplementary Material 1

## Data Availability

Extracted and synthesized studies are available as supplementary material.

## Abbreviations

SCTS: sickle cell trait screening
SCD: sickle cell disease
